# Sequencing and characterization of the complete chloroplast genome of *Sorbaria arborea* (Rosaceae)

**DOI:** 10.1080/23802359.2020.1721359

**Published:** 2020-02-06

**Authors:** Wenguang Sun, Zhe Chen, Zhenyu Lv, Zhimin Li

**Affiliations:** a School of Life Science, Engineering Research Center of Sustainable Development and Utilization of Biomass Energy, Yunnan Normal University, Kunming, China;; b Key Laboratory for Plant Diversity and Biogeography of East Asia, Kunming Institute of Botany, Chinese Academy of Sciences, Kunming, China

**Keywords:** *Sorbaria arborea* C. K. Schneider, chloroplast genome, phylogenetic analysis

## Abstract

*Sorbaria arborea* is a species which is endemic to China. We utilized next-generation Illumina sequencing technology to sequence and assemble its chloroplast genome. The 160,137 bp genome contained four main sections, including a pair of inverted repeat regions which were 26,332 bp in length, and a small single-copy region was 19,418 bp, as well as a large single-copy region, which was 88,055 bp. The genome had a GC content of 36.1% and contained 113 unique genes, including 4 rRNAs, 30 tRNAs, and 79 protein-coding genes. The phylogenetic relationship of 25 Rosaceae species was constructed based on their chloroplast genome sequences, which supported a close relationship between *S. arborea* and Amygdaloideae. This newly sequenced plastid genome provides useful information for assessing the genetic diversity and phylogenetic position of *S. arborea*.

*Sorbaria arborea* C. K. Schneider (Spiraeoideae: Rosaceae) is endemic to China and is primarily distributed in temperate and tropical mountains, especially forest margins, slopes, or stream sides at altitudes mostly ranging from 1600 to 35,000 m (Lu et al. 2003). *Sorbaria* is a small genus with nine species, two of which are endemic to China. Rosaceae is an important family for the study of taxonomy as well as systematics, because of the many economically important fruits. However, a large number of phylogenetic relationships between its genera are still in dispute (Chin et al. 2014). The complete chloroplast genome has been widely used to resolve difficult phylogenetic relationships in many plant species. In this study, we assembled the complete *Sorbaria arborea* chloroplast genome by employing high-throughput sequencing and assembly. This new plastome provides an important resource for comparative genomics and genetic conservation of this important endemic species.

Fresh leaf samples *S. arborea* were obtained from Xiaojin County (30.98472222 N, 102.69194440E), Sichuan province (voucher specimens: MCQ-081) and deposited in Herbarium of Kunming Institute of Botany (KUN), Chinese Academy of Sciences. In order to construct and sequence paired-end Illumina libraries, total genomic DNA was extracted and sent to Beijing Genomics Institute, Shenzhen. After sequencing, the resulting reads were used to construct the plastome genome using GetOrganelle (https://github.com/Kinggerm/GetOrganelle) (Jin et al. 2019) with 105 bp k-mers. First, contigs were joined together into the complete plastome using Bandage Windows v.8.1, with some manual adjustment (Wick et al. 2015). Then, the complete cp genome annotation was performed on PGA with *Rosa odorata* var. *gigantea* (GenBank: KF753637) used as a reference (Qu et al. 2019). The annotation was then curated manually using Geneious R9 v.9.0.4 (Kearse et al. 2012). Finally, a map of the genome was generated through the use of OGDRAW (https://chlorobox.mpimp-golm.mpg.de/OGDraw.html) (Greiner et al. 2019). The final chloroplast genome of *S. arborea* was deposited into GenBank (Accession number: MN901450).

The *S. arborea* chloroplast genome is 160,137 bp long and circular in shape. It exists in four major sections, including an 88,055 bp large single-copy region (54.99%), a 19,418 bp small single-copy region (12.12%), and two 26,332 bp regions which are inverted repeats (32.89%). The GC content was 36.1%. In total, the genome contained 113 genes, including 4 rRNAs, 30 tRNAs, and 79 PCGs.

The complete chloroplast genomes of 25 Roasaceae species were used to construct a phylogenetic tree with five outgroup species (Figure 1). MAFFT was then used to align all sequences (Katoh et al. 2019). We then utilized the Akaike Information Criterion in jModeltest v2.1.10 to determine the gamma rate heterozygosity and model of evolution (Darriba et al. 2012). Maximum likelihood analysis was then performed using RAxML v8.2.11 (Stamatakis et al. 2008) and the robustness of the predicted tree was assessed using 1000 bootstrap replicates with a GTR + I+G substitution model. We then used the genome sequences of the 25 Rosaceae species to reconstruct their phylogeny, which revealed a close evolutionary relationship between *S. arborea* and Amygdaloideae. This complete plastid genome should provide a useful resource for researchers examining the phylogenetic position of *S. arborea.*

**Figure 1.**
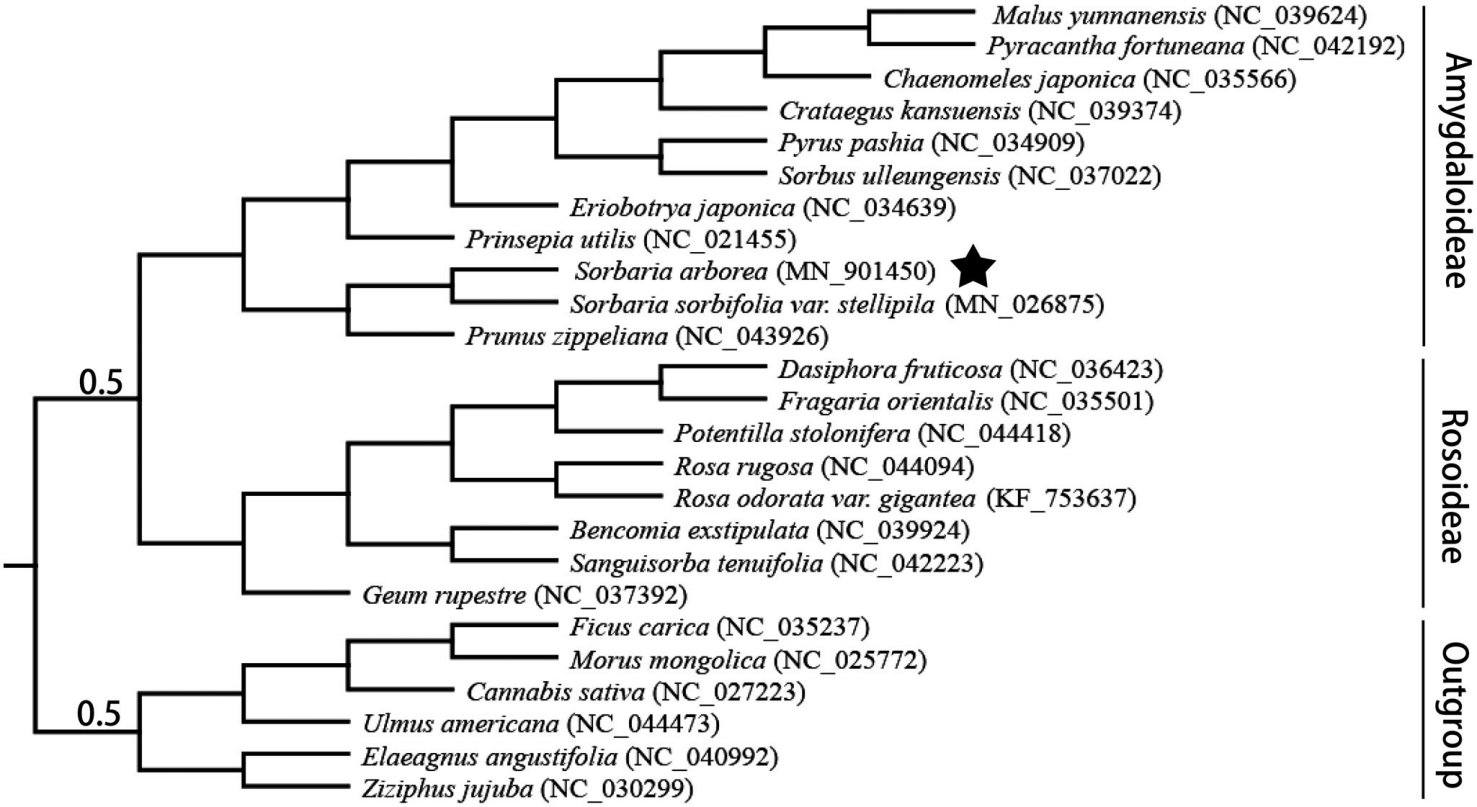
Maximum likelihood was used to determine the phylogenetic tree using 25 complete chloroplast genomes. One thousand replicates were used to determine the bootstrap values, with values less than 100% displayed beside nodes.
